# Editorial: Stem Cell Systems Bioengineering

**DOI:** 10.3389/fbioe.2021.693107

**Published:** 2021-05-07

**Authors:** Yuguo Lei, Stephanie Michelle Willerth, Tiago G. Fernandes

**Affiliations:** ^1^Department of Biomedical Engineering, Huck Life Science Institute, Pennsylvania State University, University Park, PA, United States; ^2^Division of Medical Sciences, Department of Mechanical Engineering, University of Victoria, Victoria, BC, Canada; ^3^Department of Bioengineering and iBB–Institute for Bioengineering and Biosciences, Instituto Superior Técnico, Universidade de Lisboa, Lisbon, Portugal; ^4^Associate Laboratory i4HB–Institute for Health and Bioeconomy, Instituto Superior Técnico, Universidade de Lisboa, Lisbon, Portugal

**Keywords:** engineering cell systems, stem cells, cell fate specification, bioprocessing, regenerative medicine

Stem cells hold tremendous promise for advancing the field of regenerative medicine. Stem Cell Systems Bioengineering uses engineering principles to advance the knowledge of biological systems, which ultimately contribute to the translation of new therapeutic approaches to clinical practice. The key technologies associated with this field include novel bioprocesses for the maintenance and expansion of human stem cells, as well as their differentiated progeny, and micro/nanofabrication to produce tissue-like substitutes. Other emergent topics include the development of cellular products using innovative three-dimensional (3D) cultivation systems, e.g., organoids, functional human tissue-like substitutes, controlled-release particles for programming cellular responses, novel bioprinting to generate tissues, and using artificial intelligence to model these systems. Furthermore, the development of *in vitro* tests for toxicity, cell differentiation, genomic stability of expanded cells, and biocompatibility can benefit from these scientific and technological advancements. Finally, these technologies should be compatible with Good Manufacturing Practice (GMP) conditions for eventual translation to clinical application. Accordingly, this Research Topic contains a number of contributions related to these aspects of stem cell bioengineering, including original research articles, short reviews, and reviews that cover a variety of model systems, including embryonic and induced pluripotent stem cells (iPSCs), hematopoietic stem/progenitor cells (HSPCs), mesenchymal stem cells (MSCs), and neural stem cells (NSCs).

Recent progress in the bioengineering field has allowed the manipulation of singular aspects of the cellular microenvironment. This Research Topic focused on outstanding examples of bioengineering approaches used to promote the self-organization of human cells and the production of tissue-like structure formation. Also crucial to this endeavor is the understanding of the biological system in hand, and special attention was also given to methodologies that advance our understanding of the mechanisms that control stem cell activity. Moreover, such advancements should eventually be translated to the clinic to maximize their impact. Clinical translation will require scalable processes for the maintenance and expansion of transplantable human cells, particularly novel 3D organoid systems and devices for the production of functional tissue-like substitutes.

As mentioned above, 3D cell culture has been widely used to recreate the cellular microenvironment *in vitro* and control important cell signaling cues. Consequently, this Research Topic focused on diverse aspects of 3D cell culture. Gopal et al. explored the possible combination of CRISPR Cas9 and 3D cell culture methods to enhance our understanding of the molecular mechanisms underpinning several disease phenotypes. This review outlines the possibility of bridging CRISPR Cas9 genome editing with 3D spheroid and organoid cell culture to better understand disease progression and address potential gaps to enable widespread use of these systems. Developmental biology and reproductive medicine would benefit significantly from these advancements. Artificial gametes and embryos represent a new tool for understanding human development. Zhang et al. reviewed the most recent efforts to achieve the reconstitution of the entire cycle of gametogenesis and embryo development *in vitro*. These efforts require the development of 3D culture systems capable of promoting self-organization and production of functional tissue. Silva et al. introduced a novel differentiation strategy that uses a defined medium to generate cerebellar tissue from human iPSCs. In particular, Purkinje cells, granule cells, interneurons, and deep cerebellar nuclei projection neurons were differentiated from human iPSCs and self-formed into electrically active neuronal networks *in vitro* without the need for co-culturing with rodent cells.

The examples mentioned above highlight the wide range of stimuli that can be used to guide tissue self-formation *in vitro*. NSCs are particularly responsive to biomaterial scaffolds providing appropriate mechanical and physicochemical cues that contribute to cell organization (e.g., cell alignment). Amores de Sousa et al. reported the impact of combining nanofiber alignment with functionalization of electrospun poly-ε-caprolactone nanofibers with biological adhesion motifs on the culture of NSCs. Aligned nanofibers directed NSC distribution and, in the presence of laminin and RGD-containing peptides, promoted higher cell elongation, a higher percentage of differentiated neurons, as well as significantly longer neurite development. Fang et al. also demonstrated the impact of 3D culture using graphene foams to support NSC growth and maintenance of an active proliferative state, which resulted from a reconfiguration of cell metabolism. Interestingly, recent evidence also shows that electrical stimulation improves neuronal differentiation of NSCs. Sordini et al. validated this by using electroconductive biocompatible materials for NSC culture and differentiation. Furthermore, electrical stimulation may also prove effective in promoting the differentiation of other stem cell types, like MSCs. Dawson et al. presented a theoretical framework to evaluate the influence of the applied electric field on osteogenic differentiation of MSCs. Their results showed that the differentiation rate depended on the applied electrical field, confirming other experimental findings reported in the literature.

Abecasis et al. explored another approach, based on alginate microencapsulation and suspension culture, to develop 3D human cardiac microtissues. Cell encapsulation allowed the co-culture of cardiac derivatives, including aggregates composed of cardiomyocytes, endothelial cells, and mesenchymal cells. Detailed characterization of the 3D cardiac microtissues revealed that crosstalk between different cell types and the extracellular matrix induced the maturation of iPSC–derived cardiomyocytes. Similarly, the study of the liver microenvironment has demonstrated the important roles of biochemical and biomechanical signals in regulating the progenitor cell functions that underlie liver morphogenesis and regeneration. Gentile et al. proposed an *in vitro* 3D liver spheroid model with integrated polyethylene glycol hydrogel microparticles for the local delivery of microenvironmental cues. This study demonstrated that treatment with the growth factor TGFβ1 directs differentiation of the spheroidal liver progenitor cells toward a biliary phenotype. These findings have direct implications in tissue engineering, drug testing, and our understanding of cellular metabolism. This work has critical implications in the context of the pancreas, which is of paramount importance for proper insulin secretion and maintenance of normal glycemia. In fact, it is now well-established that malfunction or destruction of β cells in the human pancreas causes diabetes. Velazco-Cruz et al. focused on this issue and reviewed recent advances in the engineering of β cells to understand and improve pancreatic cell differentiation and functional maturation. The authors summarized new differentiation strategies capable of producing advanced cellular systems with the ability to respond to glucose levels, secrete insulin, and rapidly achieve normal glycemia when transplanted into diabetic mouse models.

Scalable processes for the production of transplantable human cells and functional tissue-like substitutes are of special interest since they can significantly accelerate the translation of these products to the clinic, as these applications require significant numbers of cells. Human MSCs are a promising candidate for cell therapies given their tropism to the site of injury and secretion of trophic factors that facilitate tissue healing and/or modulate the immune response. Tsai et al. proficiently summarized the fundamental principles and concepts guiding future design of biomanufacturing systems for MSC-based therapy. Furthermore, Elanzew et al. also proposed the StemCellFactory, a modular platform that automates cell reprogramming and enables parallel derivation and expansion of human iPSC lines. This setting employs state-of-the-art cell culture techniques for optimal automated reprogramming of fibroblasts, clonal isolation, and parallel expansion of the emerging iPSCs colonies in multi-well-plates over ten passages to generate stocks of human iPSC lines.

Finally, the use of artificial intelligence and modeling to better understand differentiation and improve the production of a specific cell type was also covered in this Special Research Topic. Williams et al. examined the ability of data-driven modeling using machine learning for identifying critical experimental conditions and predicting cardiomyocyte content. The authors used data collected during human iPSC-cardiac differentiation in advanced stirred tank bioreactors to build their predictive models. Similarly, Branco et al. used a methodology based on experimental design and factorial modeling to optimize the cytokine cocktail, maximizing HSPC expansion *in vitro*. This tailored approach successfully led to an increase in HSPC productivity while enhancing our knowledge on the impact of cytokine supplementation on the HSPC yield.

In conclusion, we are proud to present our Research Topic “Stem Cell Systems Bioengineering” in Frontiers in Bioengineering and Biotechnology. We believe that all readers should experience the essence and possibilities surrounding engineering cell systems as covered in our topic. We are also optimistic that this Research Topic provides outstanding insights into the important issues of stem cell research and regenerative medicine.

## Author Contributions

All authors listed have made a substantial, direct and intellectual contribution to the work, and approved it for publication.

## Conflict of Interest

SW has a commercialization agreement with Aspect Biosystems with regards to bioprinting stem cell derived tissues. YL is a co-founder of CellGro Technologies, LLC, a company focusing on cell expansion technologies. The remaining author declares that the research was conducted in the absence of any commercial or financial relationships that could be construed as a potential conflict of interest.

